# Positive Regulation of the Antiviral Activity of Interferon-Induced Transmembrane Protein 3 by S-Palmitoylation

**DOI:** 10.3389/fimmu.2022.919477

**Published:** 2022-06-13

**Authors:** Shubo Wen, Yang Song, Chang Li, Ningyi Jin, Jingbo Zhai, Huijun Lu

**Affiliations:** ^1^ Preventive Veterinary Laboratory, College of Animal Science and Technology, Inner Mongolia Minzu University, Tongliao, China; ^2^ Key Laboratory of Zoonose Prevention and Control, Universities of Inner Mongolia Autonomous Region, Tongliao, China; ^3^ Changchun Veterinary Research Institute, Chinese Academy of Agricultural Sciences, Changchun, China

**Keywords:** interferon-inducible transmembrane proteins, S-palmitoylation, post-translational modifications, interaction, interferon-stimulated gene

## Abstract

The interferon-induced transmembrane protein 3 (IFITM3), a small molecule transmembrane protein induced by interferon, is generally conserved in vertebrates, which can inhibit infection by a diverse range of pathogenic viruses such as influenza virus. However, the precise antiviral mechanisms of IFITM3 remain unclear. At least four post-translational modifications (PTMs) were found to modulate the antiviral effect of IFITM3. These include positive regulation provided by S-palmitoylation of cysteine and negative regulation provided by lysine ubiquitination, lysine methylation, and tyrosine phosphorylation. IFITM3 S-palmitoylation is an enzymatic addition of a 16-carbon fatty acid on the three cysteine residues within or adjacent to its two hydrophobic domains at positions 71, 72, and 105, that is essential for its proper targeting, stability, and function. As S-palmitoylation is the only PTM known to enhance the antiviral activity of IFITM3, enzymes that add this modification may play important roles in IFN-induced immune responses. This study mainly reviews the research progresses on the antiviral mechanism of IFITM3, the regulation mechanism of S-palmitoylation modification on its subcellular localization, stability, and function, and the enzymes that mediate the S-palmitoylation modification of IFITM3, which may help elucidate the mechanism by which this IFN effector restrict virus replication and thus aid in the design of therapeutics targeted at pathogenic viruses.

## Introduction

When pathogens invade the host cells, pattern recognition receptors (PRRs), presenting in the host endosomes and within the cytoplasm, could recognize the microbial components as pathogen-associated molecular patterns (PAMPs) to induce an innate immune response.

In total, six PRRs have been discovered so far, including retinoic acid-inducible gene-I-like receptors (RLRs), Toll-like receptors (TLRs), NOD-like receptors (NLRs), C-type lectin receptors (CLRs), cyclic GMP-AMP synthase (cGAS), and absent in melanoma 2 (AIM2). RLRs locate in the cytoplasm, and they can recognize long or short double-stranded RNAs; TLRs sense double-stranded RNA, single-stranded RNA, double-stranded DNA, and bacterial lipopolysaccharide (1); NLRs could detect microorganisms and parasites; CLRs are a class of calcium-dependent glycol-binding proteins presenting on the surface of immune cells such as macrophages, neutrophils, and immature dendritic cells, and they could distinguish β-glucan and mannan structures in fungal cell walls; cGAS predominantly distributes throughout the cytoplasm and binds to microbial DNA (2); AIM2, a member of the HIN-200 family, mainly responses to cytoplasmic dsDNA (3).

Upon binding to PRRs, PAMPs trigger signaling cascades culminating in the secretion of numerous pro-inflammatory cytokines, including type I interferon that contributes to the host antiviral response. Type I interferon acts in both autocrine and paracrine mode by binding to its receptor to activate Janus kinase (JAK), which results in activation of the downstream STAT proteins (STAT1 and STAT2), by phosphorylating their Tyr residues. The activated STAT proteins bind to IRF9 and the trimeric complex, IFN-stimulated gene factor 3 (ISGF3), translocate into the nucleus (4). ISGF3 in the nucleus induces the transcription of hundreds of IFN- Stimulated genes (ISGs) by binding to their ISRE sequences in the nucleus. These ISGs encode various of known effector proteins with different biological characteristics. They play antiviral roles during different stages of the viral life cycle, including invasion, replication, protein translation, packaging, and release (5, 6). Recent studies on innate immune response mechanisms have suggested hundreds of IFN-stimulated genes (ISGs) that could inhibit the replication of human and animal viruses (7).

For instance, Interferon-stimulated gene 15 (ISG15), which encodes ubiquitin-like proteins, can be strongly upregulated by Type I interferon treatment or pathogen infection. It mainly regulates intracellular innate immune signal transduction, therefore inducing immune tolerance and antiviral immune response through ubiquitination (8). Another example of well-studied ISGs is oligoadenylate synthetase-like proteins (OASL). OASL are a class of protein kinases induced by double-stranded RNA and could inhibit viral protein synthesis depending on their kinase activity (9, 10). Activated OASL induce ATP hydrolysis to produce (2′-5′) oligoadenylate acid and activate endoribonuclease. Once activated, endoribonuclease degrades viral nucleic acid, thereby inhibiting viral protein synthesis and viral replication. It has been shown that down-regulating the expression of OASL restrains RIG-I signaling and promotes viral replication. Conversely, overexpression of OASL can prevent the replication of a range of viruses in a RIG-I-dependent manner and promote RIG-I mediated IFN induction (11).

Among the IGSs endowed with antiviral activity, the interferon-induced transmembrane proteins (IFITMs), especially IFITM3, are the most well-characterized due to their most potent restriction of IAV, Dengue virus, etc. IFITMs are a family of conserved small transmembrane proteins in vertebrates, expressed on cytoplasmic and endolysosomal membranes (5). The human IFITMs family contains five members located on chromosome 11, including *IFITM1*, *IFITM2*, *IFITM3*, *IFITM5* and *IFITM10* genes (12). The five homologous *IFITM* genes in chicken are located on chromosome 5. Whereas in mice, there are seven *IFITM* genes, including *IFITM1-3, IFITM5-7* and *IFITM10*. *IFITM7* is located on chromosome 16 and the other six genes are on chromosome 7. Homologous *IFITM* genes have also been found in many other species, such as fish, cattle, birds, marsupials and reptiles, implicating conserved function for IFITM proteins (12–14).

IFITM proteins are composed of five domains based on their structural characteristics. Human IFITM3 contains a hydrophobic and variable N-terminal (NTD, 1-57 aa), a hydrophobic and conserved intramembrane domain (IMD, 58-80 aa), a conserved intracellular cyclic domain (CIL, 81-104 aa), a hydrophobic transmembrane domain (TMD, 105-126 aa), and a highly variable C-terminal (CTD, 127-133 aa) (15). IMD and CIL together comprise the CD225 domain, which presents in more than 300 proteins (16).

**Figure 1:**
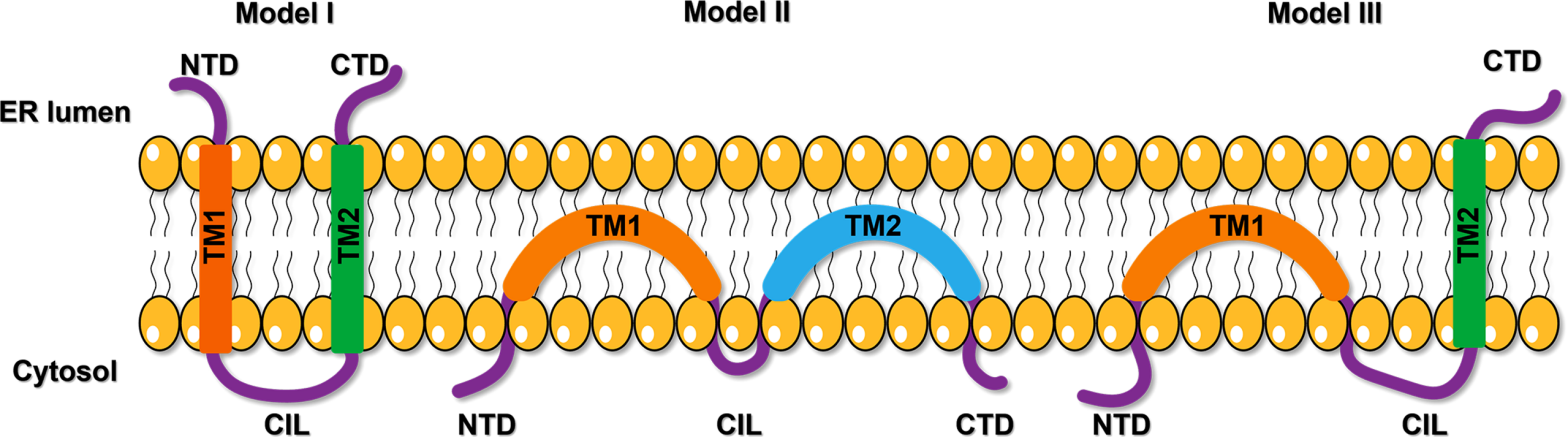
The proposed models of IFITM protein topology. The first model suggests a kind of transmembrane protein with transmembrane NTD and CTD facing extracellular. The second model shows NTD, CTD, and CIL were all located in the cytoplasm, and the two hydrophobic domains (IMD and TMD) folded back in the membrane but did not cross the membrane. The third model proposes a type II transmembrane topology, with its NTD and CIL located in the cytoplasm, while the CTD is located in the extracellular domain. NTD, N-terminal domain; CTD, C-terminal domain; TM, transmembrane domain; CIL, intracellular cyclic loop; ER, endoplasmic reticulum.

Previous studies showed that there were three models of IFITM topological structure on the membrane. Initially, IFITM was recognized as a kind of transmembrane protein with transmembrane NTD and CTD facing extracellular (**Figure 1**, Model I) (17). Subsequent studies showed that NTD, CTD, and CIL were all located in the cytoplasm, and the two hydrophobic domains (IMD and TMD) folded back in the membrane but did not cross the membrane (**Figure 1**, Model II) (18). Recently, Bailey CC et al. suggested that IFITM3 has a type II transmembrane topology, with its NTD and CIL located in the cytoplasm, while the CTD is located in the extracellular domain (**Figure 1**, Model III) (19). This model was also confirmed by some other researchers (20, 21). However, the precise topological structure of IFITM is still uncertain and needs further confirmation by crystal structure analysis.

IFITM1-3 proteins possess broad-spectrum antiviral activity against partial DNA viruses, enveloped RNA viruses, and non-enveloped RNA viruses (15, 22, 23). They can reduce the fluidity and stability of cell lipid membrane, block the fusion of the viral envelope and cell membrane to inhibit virus invasion (24). Evidence implicating IFITM3 as an innate immune protein with broad-spectrum antiviral activity accumulates rapidly. This review will summarize recent progress on the innate antiviral mechanism of IFITM3, focusing on the regulation mechanism of S-palmitoylation modification on its subcellular localization, stability, and function, and the enzymes that mediate the S-palmitoylation modification of IFITM3. This article may help elucidate how IFITM3 restricts virus replication and thus aid in developing novel therapeutic approaches to enhance the immune response against pathogenic virus infection.

## Antiviral Activities of Interferon-Induced Transmembrane Proteins

Interferon-induced transmembrane proteins (IFITM) are widely expressed and highly conserved in mammalian cells. They can be up-regulated by interferon stimulation to participate in antiviral immune responses. In recent years, small interfering RNA (15) and overexpression-based screens (25) have confirmed that IFITM1, IFITM2, and IFITM3 possess broad-spectrum antiviral effects. It has been reported that IFITM proteins, when expressed in target cells, significantly inhibit more than ten families of viruses including Alphaviridae (Semliki Forest virus, Sindbis virus) (26), Arteriviridae (Porcine reproductive and respiratory syndrome virus), Asfarviridae (African swine fever virus) (27, 28), Bunyaviridae (Rift valley fever virus, La Crosse virus, Andes virus, Hantaan virus) (29–32), Caliciviridae (Mouse norovirus) (33), Coronaviridae (SARS coronavirus) (22, 30, 34, 35), Filoviridae (Marburg virus, Ebola virus) (22, 36, 37), Flaviviridae (Dengue virus, West Nile virus, Yellow fever virus, Zika virus, Omsk hemorrhagic fever virus, Hepatitis C virus, Classical swine Fever Virus, Japanese encephalitis virus and tick-borne encephalitis virus) (23, 30, 31, 38–44), Iridoviridae (Singapore grouper iridovirus and frog iridovirus) (45–48), Nodaviridae(red spotted grouper nervous necrosis virus) (45, 46), Orthomyxoviridae (Influenza A virus) (49, 50), Paramyxoviridae (Respiratory Syncytial Virus) (51–54), Poxviridae (Vaccina virus) (55) Reoviridae (Reovirus) (56), Retroviridae (HIV-1 and Jaagsiekte sheep retrovirus) (24, 30, 57, 58), Rhabdviridae (Vesicular stomatitis virus) (57, 59, 60), Phenuiviridae (Severe fever with thrombocytopenia syndrome virus) (33).

It has been shown that IFITM1 in mammalian cells is located in the plasma membrane and early endosomes, while IFITM2 and IFITM3 are mainly expressed in the late endosomes and lysosomes (61). Due to different subcellular localization, the antiviral spectrum of the three IFITM molecules also varies. IFITM1 mainly inhibits replication of viruses that enter cells by fusing with the plasma membrane. Whereas, IFITM2 and IFITM3 mainly inhibit viruses invading cells through late endosomal and lysosomal pathways (22). Compared with IFITM1 and IFITM2, IFITM3 is considered to have the strongest antiviral activity (62). Furthermore, IFITM3, when released by the virus-infected cells into the intercellular space as a component of exosomes, could provide antiviral protection to the uninfected cells (63).

The abnormality of the *IFITM3* gene *in vivo* is involved in severe clinical symptoms caused by pathogenic viral infection. Some studies have suggested that the morbidity and mortality of *IFITM3* knockout mice infected by H3N2 or H1N1 increased (64–66). In addition, the naturally occurring single nucleotide polymorphism in IFITM3 (SNP RS12252), which is the truncated N-terminal type of IFITM3 in human may be associated with the severe outcomes following infection by IVA (64, 67), human cytomegalovirus (HCMV) (68), and human enterovirus 71 (69), as well as increased incidence and mortality caused by COVID-19 (70–72). Given the circumstance, some researchers suggested predicting the severity of COVID-19 infection among ethnic minorities based on IFITM3-rs12252 (73). Another IFITM3 SNP rs34481144 identified in the 5 ‘UTR of the *IFITM3* gene was reported to decrease the transcription level of its mRNA and the CD8^+^T cell number in the airways of influenza-infected individuals. As a result, it weakens the antiviral effect of IFITM3 and leads to severe illness in adults infected by 2009 IAV (74). These studies further attest to the physiological importance of IFITM3 in the innate immune response to pathogenic viral infections.

### The Antiviral Mechanism of IFITM3

**Figure 2:**
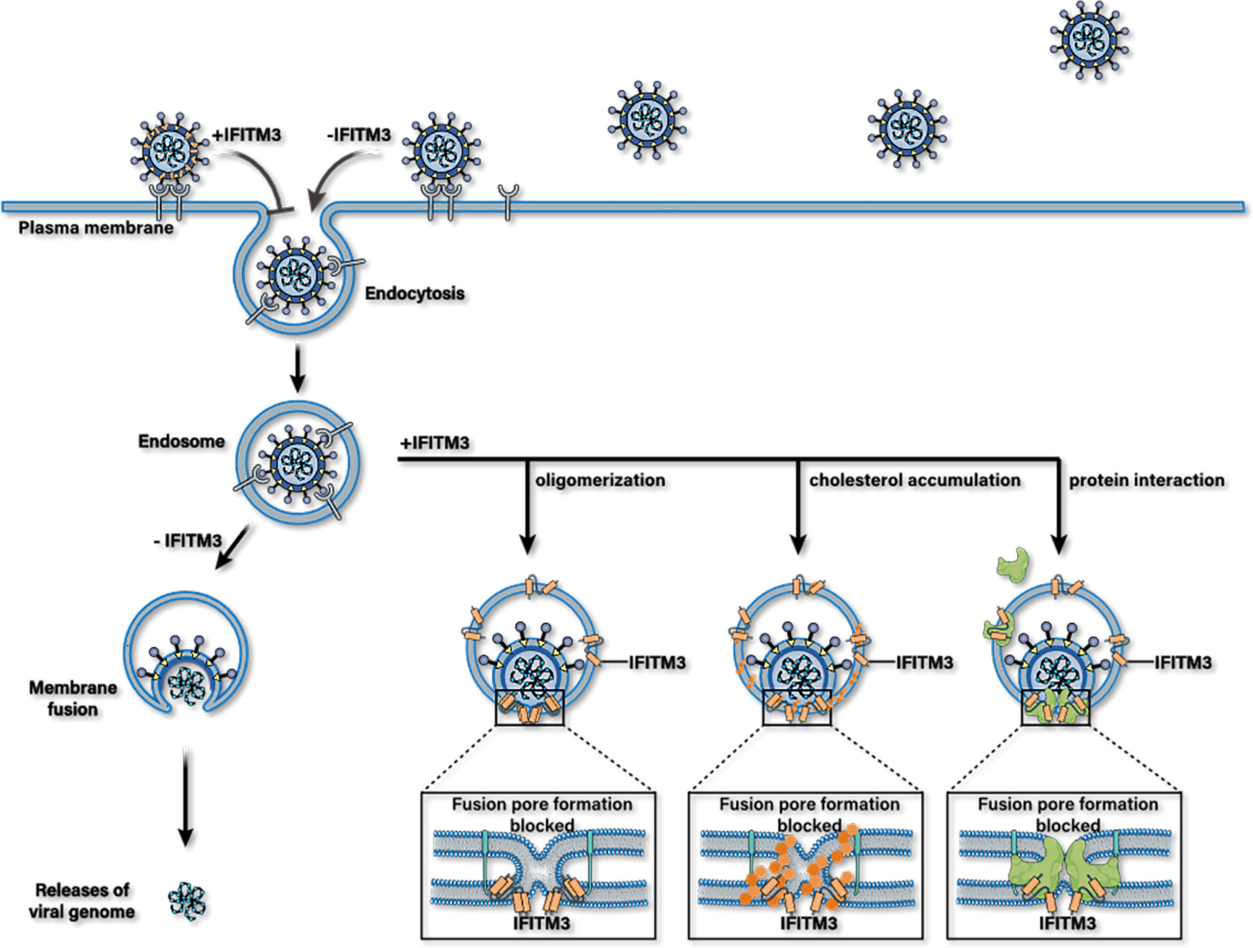
Possible antiviral mechanisms of IFITM3. IFITM3 may inhibit the release of viral protein and nucleic acid into the cytoplasm by blocking the formation of fusion pores following virus-endosome hemifusion and before forming of an enlarged fusion pore. Whereafter, it accelerates the trafficking of incoming viral particles to lysosomes for destruction. IFITM3 can bind itself to multiple host proteins, interact with cholesterol, or packages into virions to reduce their infectious activity.

IFITM3 may inhibit the release of viral protein and nucleic acid into the cytoplasm and accelerate the trafficking of incoming viral particles to lysosomes for destruction (**Figure 2**). However, the antiviral mechanism of IFITMs remains unclear till now. IFITM3 mainly plays an antiviral role in the early stage of virus infection, and at least four antiviral mechanisms are suggested based on current studies.

The first possible mechanism is that FITM3 clusters on virus-containing vesicles. This IFITM3 oligomerization increases membrane lipid order (increasing rigidity and decreasing fluidity) in cells, thus blocking fusion pore formation following virus-endosome hemifusion and before forming an enlarged fusion pore (75, 76). The overexpression of IFITM3 expands Rab7- and LAMP1-positive late endocytic compartments and accelerates the trafficking of incoming viral particles to lysosomes for destruction (77).

Moreover, IFITM3 may be incorporated into nascent virion particles during viral assembly to decrease their infectivity (39, 78, 79). The third possible mechanism is that IFITM3 may also bind itself to multiple host proteins essential to its antiviral activities. For example, a previous study showed that IFITM3 protein interacts with Vesicle-membrane-protein-associated protein A (VAPA) and inhibits its association with oxysterol-binding protein (OSBP). This effect leads to the accumulation of cholesterol in the late endosomes, and thus inhibits the cytosolic release of virions (80). Likewise, it has been reported that PRRSV was arrested in IFITM3 positive endosomes, followed by the accumulation of cholesterol in endosomes or lysosomes, resulting in obstruction of PRRSV-endosome fusion (79). However, the antiviral mechanism of IFITM3 mediated by VAPA and cholesterol has been questioned by some researchers (81–84). In addition, hABHD6A, capable of inhibiting the influenza virus by itself, has been purported to act as a cofactor required for the anti-IVA effects of IFITM3 during the diseases process (49).

Another example of a putative cofactor for IFITM3 is VCP (valosin-containing protein, also called p97). The hexametric AAA-type ATPase participates in diverse cellular processes, including endoplasmic reticulum-associated degradation (ERAD), membrane fusion, nuclear factor κB (NF-κB) activation, and chromatin-associated degradation. It has been proven to enable the antiviral function of IFITM3 by modulating its intracellular localization (85).

IFITM3 also interact with the Atp6v0b subunit of the v-ATPases within the cell to form a functional v-ATPase complex. This complex contributes to the stability of the v-ATPases and appropriate localization of clathrin, which is required for the antiviral effect of IFITM3 (86). Finally, Zinc metalloproteins STE24 (ZMPSTE24; also known as ACE1), constitutively expressed and localized to the inner nuclear membrane and cytoplasmic organelles, is an intrinsic broad-spectrum antiviral protein, which is suggested to be essential for the antiviral activity of IFITM3 through interacting with IFITM3 (37).

Finally, a recent study showed that cholesterol could change the conformation of IFITM3 in membrane bilayers and interact with S-palmitoylated IFITM3 directly. CARC domain lies on N-terminus of IFITM3, a conserved motif that mediates direct interaction between this transmembrane protein and cholesterol. Further study suggested that the CARCΔ construct of IFITM3 showed a significant loss of antiviral activity against IAV and SARS-CoV-2 infection compared with IFITM3 WT (87). In addition, cholesterol can facilitate the negative membrane curvature induced by IFITM3 resulting in increased lipid order and membrane stiffness (75). Therefore, cholesterol may play a crucial role in blocking virus fusion and the release of genetic material into the cytosol.

### The Antiviral Activity of IFITM3 Was Regulated by at Least Four PTMs

**Figure 3:**
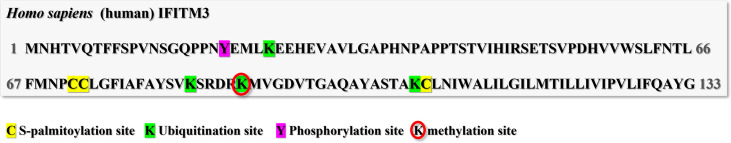
Post-translational modification sites of human IFITM3. The human IFITM3 protein was phosphorylated on tyrosine20; Ubiquitinated on Lysine24, 83, 88 and 104; methylated on Lysine88; and S-palmitoylated on cysteine71,72 and 105.

IFITM3 is a highly regulated protein with at least four PTMs occurring on multiple residues reported till now (**Figure 3**). For example, ubiquitination, the addition of the 9kDa ubiquitin polypeptide to proteins, occurring on the four lysines has been suggested to be a negative regulator of IFITM3 stability and activity by targeting the protein away from endolysosomes for degradation (18). Once all four lysines were mutated into alanine, IFITM3 became more stable and was completely located in the endosomal membrane. Meanwhile, its co-localization with endoplasmic reticulum markers also disappeared, indicating that ubiquitinated IFITM3 was recruited to the endoplasmic reticulum for degradation.

Another negative regulator of IFITM3 activity has been reported to be the protein-tyrosine kinase FYN-dependent phosphorylation on tyrosine 20 (Tyr20) (88). Mutation of Tyr20 resulted in decreased antiviral activity against vesicular stomatitis virus and enrichment of IFITM3 at the plasma membrane (88). Interestingly, cross-regulation of phosphorylation and ubiquitination of IFITM3 was observed in a previous study. It is generally expected that IFITM3 phosphorylation may enhance its ubiquitination level because phosphorylation normally serves as a signal for E3 ubiquitin ligases. However, the opposite was observed in that IFITM3 ubiquitin was attenuated by phosphorylation (89). How ubiquitin and phosphorylation co-regulate the antiviral effect of IFITM3 deserves further study.

Finally, single lysine (Lys88) methylation of IFITM3 mediated by SET7 has also been described to negatively regulate IFITM3 antiviral activity (90). In this study, methylation of IFITM3 was up-regulated by SET7 overexpression leading to a loss of antiviral activity. In contrast, knockdown of SET7 decreased IFITM3 methylation and resulted in enhanced IFITM3 antiviral activity against influenza virus and vesicular stomatitis virus.

Except for the three PTMs, S-palmitoylation of IFITM3 is the only one that can positively regulate its antiviral activity by multiple mechanisms discussed in the following.

## Protein S-Palmitoylation

Palmitoylation is the posttranslational process by which a 16-carbon palmitic acid is covalently attached to eukaryotic and viral proteins. It is catalyzed by a family of proteins known as DHHC acyltransferases. The most common forms of palmitoylation are S-palmitoylation and N-palmitoylation. S-palmitoylation, a reversible modification, occurs on cysteine residues *via* a thioester bond and is commonly found in most palmitoylated proteins. N-palmitoylation reactions occur on the amino terminus or the epsilon amino group of lysine.

Recently, improved proteomic of cellular proteins has identified tens to hundreds of proteins as substrates for S-palmitoylation. Many lines of evidence suggest that S-Palmitoylation is implicated in various physiological processes, including intracellular trafficking, the activity of ion channels, localization of neuronal scaffolding proteins Ras signaling, and host-pathogen interactions (91–95). As a posttranslational modification, S-palmitoylation was first reported in 1979 (96). However, the enzymes that catalyze protein S-palmitoylation were not discovered until 2002 in yeast (97, 98). A family of low-abundance and polytopic eukaryotic integral membrane enzymes responsible for modifying proteins with palmitate on the cytoplasmic face of cellular membranes is DHHC-palmitoyl transferase. They are so named because they share a signature DHHC (Asp-His-His-Cys) motif, the catalytic center of the enzyme, within a cysteine-rich domain.

Many intracellular soluble and membrane proteins can undergo S-palmitoylation modification, generally occurring in intracellular-membrane contact. S-palmitoylation modifies the hydrophobicity of proteins, affects their membrane-binding properties, intracellular sorting, stability, and thus regulates a series of cellular physiological and pathological processes.

### S-Palmitoylation and Viral Infection

It has been suggested that S-palmitoylation modification is also involved in the host defense against pathogens. Some pathogens use palmitoyl transferase of host cells to modify their virulence proteins to enter host cells (95, 99). In 1979, S-palmitoylation of Sindbis virus envelope glycoprotein and vesicular stomatitis virus glycoprotein G were firstly identified (96, 100). With the development of technologies, many other viral proteins have also undergone S-palmitoylation modification as well. Previous studies have shown that S-palmitoylation of influenza virus HA contributes to the recruitment of the viral protein to membrane lipid raft, the budding process of new virus particles, and the fusion of virus particles and membrane to promote virus invasion (101–103). Recent studies have shown that S-palmitoylation modification of HA regulates the membrane curvature and promotes the interaction between HA and matrix protein (M1) during viral particle assembly (104), which promotes the expansion of fusion pores (102). The highly conserved amphiphilic helical region of M2 is also S-palmitoylated to regulate the membrane curvature, promote the separation of the virion from the membrane, and virion release (105, 106).

### S-Palmitoylation and Innate Immunity

Chesarino et al. found that S-palmitoylation of TLR2 (a member of the TLR family) mediated its membrane localization, NF-κB activation and inflammatory factor release. Once inhibited, its response to all microbial ligands were restrained. Palmitoyl transferases ZDHHC2, 3, 6, 7, and 15 are responsible for the TLR2 S-palmitoylation (107).

In 2016, Mukai’s lab proved that S-palmitoylation of interferon gene stimulator protein (STING) affects its ability to regulate innate immune signals. STING was S-palmitoylated in the Golgi and is restricted to the trans-Golgi network (TGN). The general S-palmitoylation inhibitor 2-BP can selectively inhibit STING-mediated cytoplasmic DNA sensing and its downstream interferon response. In addition, a STING Cys88/91Ser double mutant that significantly reduced S-palmitoylation could not induce STING-dependent host defense after infection with DNA viruses, and palmitoyl transferases ZDHHC3, 7, and 15 are responsible for STING modification (108).

Studies have shown that S-palmitoylation of Rac1(the Rho family Small Guanosine triphosphatase SE) inhibits the ubiquitination activation of E3 ubiquitin ligase TRIM31 on MAVS, thereby negatively regulating the RLR signaling pathway. However, it remains unclear which ZDHHC protein is responsible for the S-palmitoylation of Rac1 (109).

Recently, Lu and colleagues suggested that targeting NOD1 and NOD2 to bacterial-containing endosomal membranes requires them to be S-palmitoylated by ZDHHC5. They identified pathogenic bacterial-derived peptidoglycan components in the cytoplasm that promote intracellular NOD1/2-mediated immune responses, including autophagy and release of inflammatory factors (110). However, the precise mechanism that ZDHHC5 recruits bacteria to the entry site remains unclear.

It was found that S-palmitoylation of cysteine residues at position 463 near the cytoplasmic end of the IFN AR1 transmembrane region is necessary for phosphorylation activation of STAT1 and STAT2 (downstream of IFNAR1). Blocking S-palmitoylation of IFNAR1 inhibits the nuclear translocation of STAT and thus affects the transcription of ISGs. However, S-palmitoylation of IFNAR1 has no effect on its *in vivo* endocytosis, intracellular distribution, stability on the cell surface, and the formation of IFNAR1-IFNAR2 heterodimer (111).

## Mechanism of S-Palmitoylation to Regulate the Antiviral Capacity of IFITM3

To date, S-palmitoylation is believed to be essential for the antiviral activity of IFITM3, but the research on the molecular mechanism of how S-palmitoylation regulates its antiviral activity is relatively slow. One of the main reasons is that protein S-palmitoylation was originally studied using radioactive palmitate metabolic labeling methods. However, this approach has the defects of limited sensitivity and poor security. New technologies, such as acy-biotinyl exchange and click-chemistry that allow S-palmitoylation sites to be experimentally-determined have greatly promoted the study of this lipid modification (112). Currently, the research progresses of IFITM3 S-palmitoylation were listed in **Table 1**.

**Table 1:** Research progresses of IFITM3 S-palmitoylation.

Year	Progress	Reference
2010	S-palmitoylation of IFITM3 was first identified and this lipid modification controls its clustering in membrane compartments and anti-IVA activity.	(113)
2012	IFITM3 had an inherent affinity for cell membranes, enhanced by hydrophobic S-palmitoylation	(18)
2013	a C72A mutation made human IFITM3 more centralized intracellular distribution	(16)
2016	Acyl-PEG exchange (APE) was developed, and dually S-palmitoylated IFITM3 at Cys72 and Cys105 was suggested to be the most active isoform in mammalian cells	(114)
2017	More than half of the ZDHHCs were capable of increasing IFITM3 palmitoylation with ZDHHCs 3, 7, 15, and 20 having the greatest effect	(115)
2019	S-palmitoylation of IFITM3 at Cys72 is important for its rate of trafficking to IAV particles during infection	(116)
2020	S-palmitoylation has no effect on the subcellular localization of swine IFITM3;	(38)
	S-palmitoylation on the three cysteine residues were all crucial for sIFITM3 to restrict PRRSV replication;	(79)
	All cysteine mutants of microbat IFITM3 relocalized to perinuclear Golgi-associated sites;	(117)
	S-palmitoylation of IFITM3 mediated by ZDHHC1 was indispensible for keeping it from lysosomal degradation.	(39)
2021	modulation of IFITM S-palmitoylation levels and cholesterol interaction may influence host susceptibility to different viruses;	(87)
	S-palmitoylation also enhanced the antiviral activity of IFITM3 by modulating its conformation and interaction with lipid membranes.	(62)

**Figure 4:**
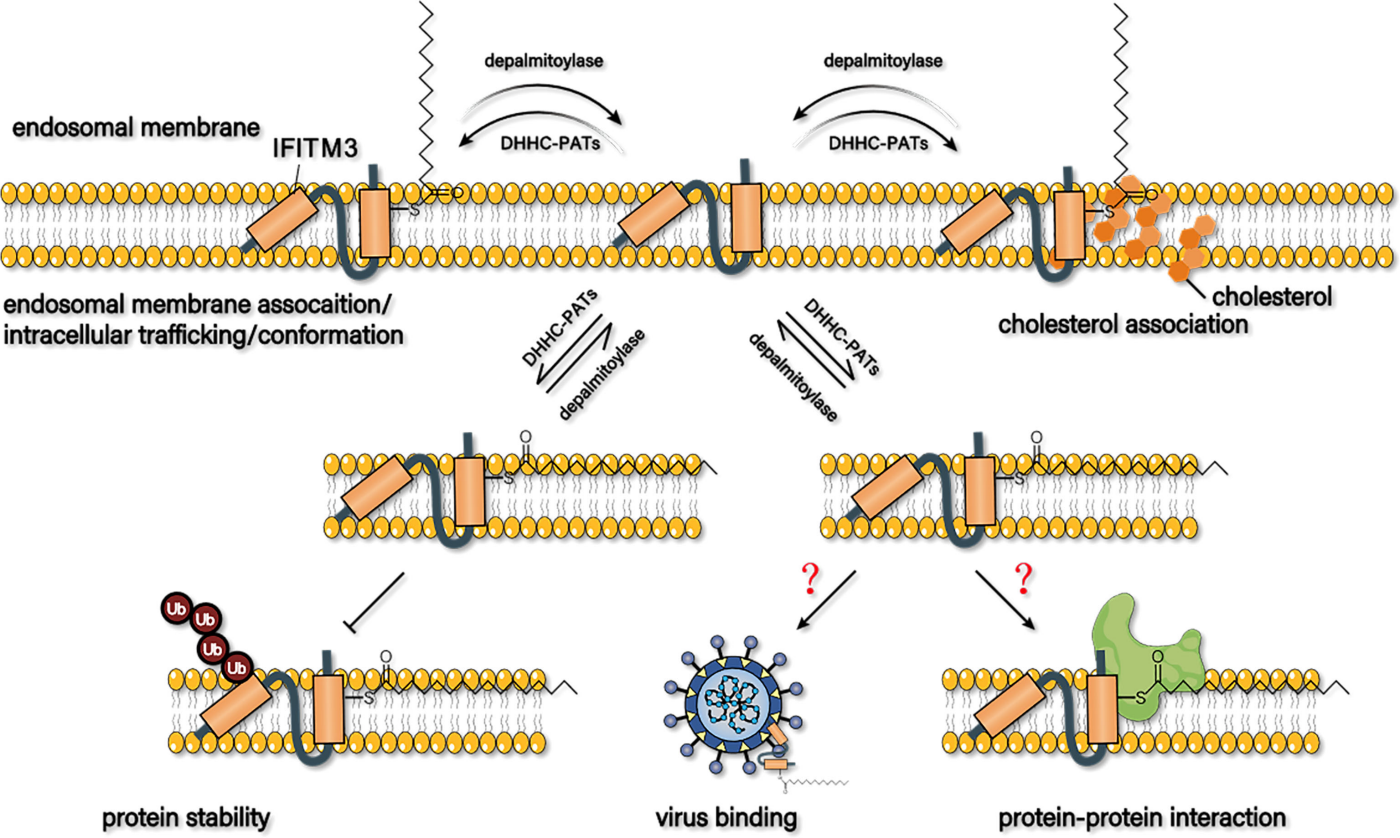
Possible mechanism of s-palmitoylation to regulate the antiviral capacity of IFITM3. S-palmitoylation may affect its membrane proportion, trafficking to virions, stability, interaction with cholesterol, and conformation to regulate the antiviral activity of IFITM3. Whether S-palmitoylation affects the binding of IFITM3 to virus particles or its interaction with host proteins has not been reported yet.

Based on the previous findings, S-palmitoylation of IFITM3 is essential for its antiviral effect, subcellular location, stability, virion trafficking, interaction with cholesterol, and molecular conformation (**Figure 4**).

### S-Palmitoylation Affects the Subcellular Location of IFITM3

S-palmitoylation of IFITM3 was first identified by Yount and his colleagues (113) through large-scale profiling of fatty-acylated proteins in the mouse DC line DC2.4 using palmitic acid chemical reporter ALK-16 and CuAAC. Further study revealed that all the three cysteine residues at positions Cys71, Cys72, and Cys105 of IFITM3 were S-palmitoylated. Bioinformatics analysis showed that the S-palmitoylated Cysteine residues are conserved in IFITM isoforms in most vertebrates, suggesting an added layer of regulation of protein S-palmitoylation in innate immunity. Moreover, it was observed that the S-palmitoylation of IFITM3 on membrane-proximal cysteines enhanced its clustering in the membrane compartments. Whereas, the mutation of the three cysteine residues to alanine eliminated the S-palmitoylation of IFITM3, resulting in its spot-like distribution in the endoplasmic reticulum. Additionally, wild-type IFITM3 (WT IFITM3), primarily membrane-associated, also present at lower levels in the cytoplasmic portion. In contrast, IFITM3-PalmΔ mutants are less membrane-associated, suggesting that IFITM3 has an inherent affinity for cell membranes, enhanced by hydrophobic S-palmitoylation (18).

Furthermore, it has been suggested that BODIPY-labeled IFITM3 Cys-to-Ala mutants showed a subcellular localization similar to WT human IFITM3 (116). Meanwhile, a confocal experiment revealed that sIFITM3-PalmΔ (C71,72,105A) was co-located with early/late endosomes and lysosomes in MARC-145 sIFITM3-PalmΔ-Flag cells, consistent with WT IFITM3 (79). Subcellular location change of sIFITM3-PalmΔ was not observed in PK15 as well (38).

Another study proved no change in subcellular location for C71A and C105A, but a more centralized intracellular distribution for C72A compared with WT human IFITM3 (16). Besides, all cysteine mutants of microbat IFITM3 relocalized to perinuclear Golgi-associated sites (117). Together, these studies indicate that S-palmitoylation regulates the subcellular location of human, murine, swine and microbat IFITM3 in different manners.

### S-Palmitoylation Affects the Antiviral Activity of IFITM3

Mounting evidence showed that IFITM3 S-palmitoylation was crucial for its antiviral effect. For example, the S-palmitoylation-deficient, triple cysteine-to-alanine IFITM3 mutant significantly weakened its anti-IAV activity (113). A consistent result was observed in another study, in which the antiviral activity of the IFITM3 S-palmitoylation deficient mutant against H1N1 influenza virus (type A, PR8 strain) was significantly reduced (18).

As the analysis of S-palmitoylation levels of endogenous IFITM3 is in need to better elucidate the association between S-palmitoylation and IFITM3 antiviral activity, a mass-tag labeling method termed acyl-PEG exchange (APE) was developed (114). The APE analysis combined with fatty acid metabolic labeling showed that S-palmitoylation occurred in most endogenous IFITM3 in IFN-stimulated cells, and Cys72 was the predominant S-palmitoylation site. Indeed, after the mutation of Cys72 into alanine, the antiviral effect of IFITM3 decreased significantly. Although Cys71 of IFITM3 is highly conserved in mice and humans, its mutation to Ala is reported to have no significant effect on the S-fatty level and antiviral effect of IFITM3. The effect of Cys105 S-palmitoylation on the antiviral activity of IFITM3 varies in different IFITM isoforms, expression levels, and cell types, since mutation of this Cys residue hampers the antiviral activity of IFITM3 in mice but does not affect the activity of human IFITM3.

It is also proved that the mutant of Cys72 to Ala significantly reduced its antiviral activity against IAV, while the IFITM3 mutant of Cys71 and Cys105 to Ala retained more activity (116). These results suggest that the antiviral activity of IFITM3 is directly related to the level of S-palmitoylation, and the double S-fatty acylation of IFITM3 on Cys72 and Cys105 may be the most active subtype in mammalian cells. However, when any one of the three cysteines was mutated to alanine, the anti-PRRSV activity of sIFITM3 was almost completely lost (79), indicating that S-palmitoylation on the three cysteine residues were all crucial for sIFITM3 in PRRSV restriction.

### S-Palmitoylation Affects the Trafficking of IFITM3 to Virions

S-palmitoylation affects the trafficking of IFITM3 to virions as well. Compared with WT IFITM3 or Ala mutants at Cys71 or Cys105, trafficking and co-localization of IFITM3-C72A mutants with quenched DID-IAV particles was significantly reduced by about 20% through site-specific fluorescent labeling and live-cell imaging analysis. Meanwhile, the trafficking of IFITM3-C72A mutants to DID-IAV particles was also delayed (116). These results suggest that S-palmitoylation at Cys72 is important for the IFITM3 trafficking to IAV particles during infection.

### S-Palmitoylation Affects the Stability of IFITM3

S-palmitoylation also affects the stability of IFITM3. It has been revealed that FITM3 protein abundance was significantly decreased in cells treated with the general protein palmitoylation inhibitor (2-brompalmitate, 2-BP), and the inhibition effect is dose- and time-dependent, but IFITM3 mRNA levels were not affected. The half-life of IFITM3 protein in 2-BP treated cells was significantly reduced to 1.5 h vs 5 h in control cells. Additionally, exogenous IFITM3ΔPalm mutants were degraded rapidly, with a half-life of 2.9 h compared with 7.9 h for WT IFITM3. Moreover, 2-BP-induced IFITM3 degradation in A549 and HCT116 cells can be significantly blocked by lysosome pathway inhibitor (leupeptin and bafilomycin A1) rather than ubiquitin-proteasome pathway inhibitor (MG132), indicating that IFITM3 may be degraded in a lysosome pathway (not ubiquitin-proteasome pathway) when its S-palmitoylation was restrained (39). Nonetheless, IFITM3 PalmΔ mutants were reported to be ubiquitinated effectively in a previous study (114). And it has been suggested that ubiquitinated IFITM3 can be recruited to an ER-proximal site for degradation (18, 89, 118). Therefore, whether the ubiquitin-proteasome pathway is involved in S-palmitoylation deficit-induced IFITM3 degradation needs to be further elucidated.

### S-Palmitoylation Affects the Interaction Between IFITM3 and Cholesterol

Of note, the S-palmitoylation level of IFITM2 was significantly lower than IFITM3 by metabolic labeling analysis. High levels of S-palmitoylation enhanced IFITM3 interactions with cholesterol and inhibited viruses like IAV and SARS-CoV2. While with lower levels of S-palmitoylation and fewer interactions with cholesterol, IFITM2 showed more efficient anti-EBOV activity, indicating that S-palmitoylation may inhibit cholesterol-dependent viruses by regulating the interaction between IFITM3 and cholesterol (87).

### S-Palmitoylation Affects the Conformation of IFITM3

Compared with S-palmitoylation-deficient IFITM3, Cys72-maleimide palmitate, a reasonable substitute for S-palmitoylation modification, showed a significant structural change both locally and in the disordered N-terminal regions. Meanwhile, the disrupted AH1 region of S-palmitoylation-deficient IFITM3 was stabilized and its association with the membrane bilayer was increased by adding maleimide palmitate to Cys72 in a flotation assay. These results suggest that lipidation can change the biophysical conformation of IFITM3. As the disruption of AH1 attenuates the antiviral activity of IFITM3, it is suggested that the S-palmitoylation at Cys72 directly enhances the antiviral activity of IFITM3 by stabilizing AH1 (62). Besides, stabilization of IFITM3 AH1 by S-palmitoylation of Cys72 may also explain the subcellular distribution of IFITM3 during infection.

### ZDHHCs Are Implicated in S-Palmitoylating IFITM3

Although many substrate proteins of ZDHHC transferase have been identified, the mechanism of how transferase recognizes its target protein remains unclear. The distribution of each specific ZDHHC protein in different subcellular locations affects the type of substrate protein. Only a few substrate/ZDHHC pairings have been identified among the hundreds of known S-palmitoylated proteins. For example, ZDHHC9 and ZDHHC17 can only S-palmitoylate HRas and SNAP25, whereas ZDHHC14 has PAT activity toward PSD93 (30).

A variety of ZDHHC proteins are capable of S-palmitoylating IFITM3. To identify the enzyme that performs S-palmitoylation modification for IFITM3, Temet et al. constructed rodent ZDHHC1-23 expression plasmids for overexpression screening. Results showed that ZDHHC1, 2, 5, 6, 9, 14, 23, 24, and 25 could up-regulate IFITM3 S-palmitoylation by 1.7-3 times, whereas ZDHHC3, 7,15, and 20 increased the S-palmitoylation level of IFITM3 by more than 3 times. Importantly, it was reported that the three cysteine residues of IFITM3 protein were not completely modified by S-palmitoylation, which explains why a class of ZDHHC proteins can enhance IFITM3 S-palmitoylation level (115).

Wang et al. reported that p53 up-regulated ZDHHC1 expression and S-palmitoylation level of IFITM3. Overexpression of ZDHHC1 increased the S-palmitoylation level of exogenous IFITM3 and the protein expression level of endogenous IFITM3. The ZDHHC1 mutant with DHHC deficiency could still increase the S-palmitoylation level of exogenous IFITM3, but not the protein expression level of endogenous IFITM3. Meanwhile, ZDHHC1 could not up-regulate endogenous IFITM3 protein expression in the presence of 2-BP. Similar to wild-type ZDHHC1, the ZDHHC1 DHHCΔ mutant can co-precipitate with exogenous IFITM3, suggesting that ZDHHC may interact with IFITM3 independently of the DHHC region and modify it with S-palmitoylation. This study raises the question whether other regions besides DHHC are involved in ZDHHC1 palmitoyltransferase activity (39).

In total, 13 ZDHHCs have been revealed to enhance S-palmitoylation of IFITM3, including 1, 2, 3, 5, 6, 7, 9, 14, 15, 20, 23, 24, and 25. Only ZDHHC1 and ZDHHC20 can enhance the antiviral activity of IFITM3, and further study showed that ZDHHC20 and IFITM3 are co-localized in lysosomes. By contrast, ZDHHC3, ZDHHC7, and ZDHHC15 are co-localized around the nucleus with IFITM3, suggesting that the subcellular localization where hIFITM3 was S-palmitoylated may affect its antiviral activity (115). As ZDHHC2, 5,6,9,14,23,24,25 can also enhance the S-palmitoylation level of IFITM3, it’s interesting to explore where the eight ZDHHCs colocalize with IFITM3 and whether these enzymes can enhance its antiviral effect.

## Conclusions and Perspectives

In summary, S-palmitoylation of IFITM3 is essential for its antiviral effect, subcellular localization, stability, virion trafficking, interaction with cholesterol, and molecular conformation. IFITM3 can bind directly to the surface of the virus particle to reduce its infective activity or interact directly with host proteins to play an antiviral role. However, whether S-palmitoylation affects the binding of IFITM3 to virus particles or its interaction with host proteins has not been reported yet. This topic is worthy of further research, which could help to reveal the molecular mechanism of how S-palmitoylation regulates the antiviral effect of IFITM3. Furthermore, all the three cysteines of IFITM3 in murine and humans can be palmitoylated, and Csy72 is the most important palmitoylated site, followed by Csy105. In contrast, The S-palmitoylated modification of Csy71 alone seems to have the least effect on its biological function. However, it has been shown that anyone mutation of the three cysteines to alanine can almost completely abolish the anti-PRRSV activity of porcine IFITM3, suggesting that the dually S-palmitoylated IFITM3 at Cys72 and Cys105 may not necessarily be the most active isoform in mammalian cells.

In this review, we have primarily focused on the classical and novel progress concerning the antiviral mechanism of IFITM3 and the mechanism of how S-palmitoylation regulates its antiviral activity. The role of PTM of proteins involved in regulating of cellular antiviral activity has been a hot topic. Despite recent advances in understanding the mechanism of S-palmitoylated proteins in immune response, little is known about the regulation of natural immune response. Therefore, how S-palmitoylation modification regulates natural immune response could be a research focus in the future. Of note, S-palmitoylation modification participates in virus replication and infection and the regulation of antiviral effect mediated by IFITM3, but the mechanisms of how the host balances these two effects await further elucidation.

As is shown in many studies, the catalytic center of the ZDHHC family is the highly conserved DHHC domain. However, the exogenous ZDHHC1 mutants deficient in the HDDC region can enhance S-palmitoylation of IFITM3. It’s worthy of exploring whether other catalytic dominants exit in ZDHHC protein. Moreover, emerging evidence has revealed the interplay between S-palmitoylation and other PTMs. For example, inhibition of S-palmitoylation modification of IFITM3 can significantly up-regulate its ubiquitination level. What effect does S-palmitoylation modification have on the other two posttranslational modifications (methylation and phosphorylation) of IFITM3? The precise molecular mechanisms that govern the synergistic effect of PTMs deserves further exploring.

In general, S-palmitoylation regulates the antiviral activity of IFITM3 through influencing its membrane proportion, trafficking to virions, stability, interaction with cholesterol, and conformation. Future studies are still in need to clarify the regulation mechanism of S-palmitoylation on IFITM3.

## Author Contributions

Conceptualization: CL and NJ. Writing-original draft preparation: SW and YS. Writing-review: CL, and YS. Figures: SW. Supervision: NJ, JZ, and HL. Funding acquisition: SW, JZ, and HL. All authors contributed to the article and approved the submitted version.

## Funding

This work was financially supported by the Open Funding Project of Brucellosis Prevention and Treatment Engineering Research Center of Inner Mongolia Autonomous region [No. MDK2021078, MDK2019082], Doctoral Funding of Inner Mongolia Minzu University [No. BS584, BS583], and Key Research and Development Program in Inner Mongolia Autonomous Region [No. 2021ZD001301, 2019ZD006].

## Conflict of Interest

The authors declare that the research was conducted in the absence of any commercial or financial relationships that could be construed as a potential conflict of interest.

## Publisher’s Note

All claims expressed in this article are solely those of the authors and do not necessarily represent those of their affiliated organizations, or those of the publisher, the editors and the reviewers. Any product that may be evaluated in this article, or claim that may be made by its manufacturer, is not guaranteed or endorsed by the publisher.
